# Responses of Massachusetts hospitals to a state mandate to collect race, ethnicity and language data from patients: a qualitative study

**DOI:** 10.1186/1472-6963-10-352

**Published:** 2010-12-31

**Authors:** Selena Jorgensen, Ruth Thorlby, Robin M Weinick, John Z Ayanian

**Affiliations:** 1 Department of Health Care Policy, Harvard Medical School, Boston, MA, USA; 2 Nuffield Trust, London, UK; 3 RAND Corporation, Arlington, VA, USA; 4 Division of General Medicine, Brigham and Women's Hospital, Boston, MA, USA

## Abstract

**Background:**

A Massachusetts regulation implemented in 2007 has required all acute care hospitals to report patients' race, ethnicity and preferred language using standardized methodology based on self-reported information from patients. This study assessed implementation of the regulation and its impact on the use of race and ethnicity data in performance monitoring and quality improvement within hospitals.

**Methods:**

Thematic analysis of semi-structured interviews with executives from a representative sample of 28 Massachusetts hospitals in 2009.

**Results:**

The number of hospitals using race, ethnicity and language data internally beyond refining interpreter services increased substantially from 11 to 21 after the regulation. Thirteen of these hospitals were utilizing patient race and ethnicity data to identify disparities in quality performance measures for a variety of clinical processes and outcomes, while 16 had developed patient services and community outreach programs based on findings from these data. Commonly reported barriers to data utilization include small numbers within categories, insufficient resources, information system requirements, and lack of direction from the state.

**Conclusions:**

The responses of Massachusetts hospitals to this new state regulation indicate that requiring the collection of race, ethnicity and language data can be an effective method to promote performance monitoring and quality improvement, thereby setting the stage for federal standards and incentive programs to eliminate racial and ethnic disparities in the quality of health care.

## Background

A growing body of research has documented the pervasive nature of disparities in health outcomes for racial and ethnic minorities within the US[1-4]. The Institute of Medicine's (IOM) 2003 report, *Unequal Treatment: Confronting Racial and Ethnic Disparities in Health Care*, highlighted the need to transition from describing disparities to developing methods by which they can be reduced[2]. The standardized collection of data on patients' race, ethnicity, and preferred language has been widely recognized as a necessary step to improve the quality of care by developing effective interventions to reduce disparities[4-10]. In 2009 the IOM strongly recommended that health-care organizations and the federal and state governments accelerate efforts to collect these data[4].

In 2006 Massachusetts adopted a novel approach to the collection of race and ethnicity data, building on efforts by the city of Boston. The Massachusetts Division of Health Care Finance and Policy (DHCFP) issued new regulations requiring all acute-care hospitals to collect patients' self-reported race, ethnicity and preferred language with a standardized approach (Additional file 1), including an expanded list of 33 ethnic groups[11]. Hospitals were required to begin reporting these data to the state in April, 2007. The Massachusetts Hospital Association (MHA) provided training and support materials [12] and sponsored four regional training sessions for hospitals in the fall of 2006 to explain the requirements.

Early piloting of the proposed regulation helped DHCFP anticipate potential problems and concerns from patients and staff that might be encountered with widespread adoption of the regulation[13]. Hospital staff and patients voiced concerns regarding the importance of collecting data, how the data would be used, and the distinction between race and ethnicity. Determining how best to ask about Hispanic/Latino ethnicity was a particularly challenging issue in the development of the regulation, resulting in a question about Hispanic/Latino ethnicity followed by a separate question about race. Resources for modifying information technology systems and training staff were a significant concern. Information systems needed reprogramming to handle expanded ethnicity categories and multiple response options. Registration staff required training to obtain the data in a standardized manner and to address concerns from patients. Some hospital staff were also frustrated by a perceived lack of direction from the state regarding the purpose of collecting these data.

The objectives of this qualitative study were to explore these concerns two years after implementation and to assess the impact of the state regulation. To determine how the regulation has influenced hospitals' uses of race, ethnicity, and language data in performance monitoring and quality improvement, we conducted semi-structured interviews with executives from a representative sample of Massachusetts hospitals.

## Methods

### Study Subjects

From the list of 78 acute-care hospitals identified by the Massachusetts Department of Public Health, we selected a representative sample of 56 hospitals (based on resources available for study interviews) stratified by number of beds (< 100, 100-299, ≥300), location (6 regions of the state), and academic affilation (teaching or non-teaching). In July, 2009, letters requesting participation in a study about the state regulation were sent to the chief executive officers (CEOs) of the 41 health systems representing the 56 hospitals in the sample. For hospitals that did not respond to the initial letter, at least 5 attempts were made to contact them by telephone and email over a three-month period. Eighteen of the 41 health systems agreed to participate, representing 28 (50%) of the 56 sampled hospitals. Each CEO was invited to select up to three senior executives from the following areas: patient access and registration (8); community, diversity and disparities (7); quality, safety and performance (6); information technology systems (4); and finance (3). Oral informed consent was obtained from each respondent prior to the interview. The study protocol was approved by the human studies committee of Harvard Medical School.

### Data Collection

The lead author conducted the interviews by telephone with a semi-structured interview guide that included both closed- and open-ended questions developed by the research team (Additional file 2). Questions related to how the regulation affected data collection within hospitals, how staff and patients responded to these changes, and how hospitals were using data on patients' race, ethnicity and language. Interviews were electronically recorded and transcribed.

### Data Analysis

Interviews were analyzed using MaxQDA qualitative analysis software program (Version 2007; Marburg, Germany), which facilitates coding and comparison of discrete sections of text. Transcripts were reviewed by three members of the research team for themes (SJ, RT, JZA). Each member independently developed a list of themes which were reviewed for consistency. Hospital responses and receptiveness to the mandate were stratified by a set of pre-determined hospital characteristics including size, region, teaching status, and Disproportionate Share Hospital status (defined as having a majority of patient charges attributed to Medicare, Medicaid, other government payers and free care) as specified by the Massachusetts DHCFP[14].

## Results

### Study Cohort

The 28 participating hospitals represented 45.4% of all inpatient discharges in Massachusetts in 2008. Descriptive characteristics of these hospitals are presented in Table 1.

**Table 1:** Characteristics of Respondent Hospitals

Characteristic	Respondents(n = 28)	All Hospitals (n = 78)
**Region**		
**Boston**	5	13
**Metrowest**	9	16
**Northeast/Southeast**	6	27
**Central/Western**	8	22
**Number of beds**		
**≥300**	7	16
**100-299**	16	38
**< 100**	5	24
**Residency training programs**		
**Yes**	15	21
**No**	13	57
**Disproportionate Share Hospital**		
**Yes**	5	19
**No**	23	59

### Data Collection and Use Prior to the State Mandate

Of the responding hospitals, all five located within Boston and two outside the city were directly involved in the efforts of the DHCFP to develop the regulation. Representatives of these seven hospitals and one other hospital were generally very supportive of the regulation and noted it was perceived very favorably within their institutions. Executives of 15 hospitals did not convey a strong reaction for or against the regulation. Representatives of the five remaining hospitals expressed reservations about the mandate as "one more thing" the state was requiring of them. However, three of these representatives still acknowledged the importance of collecting these data, and only one hospital executive stated that the data were not relevant to his hospital's largely white, English-speaking patient population.

Over 89% of US hospitals collect race data[4]. All 28 hospitals in our study were collecting race data prior to the regulation, using categories specified by the federal Office of Management and Budget[15]. However, all respondents stated that the process of data collection prior to the regulation was not standardized and generally yielded unreliable data. Two hospitals collected patients' self-reported data prior to the regulation, while the remaining 26 relied upon staff observation or a combination of both methods. Only two hospitals had collected ethnicity data beyond a single question regarding Hispanic ethnicity. Whether or not data were even collected often depended on the individuals working in patient registration.

*Besides white, the most frequently answered category was 'other,' which meant that people were not being even nuanced between black... and Hispanic*. (Medium teaching hospital)

*Our intake forms for registration had these prompts on them, but it was sensitive at that time so the individuals collecting that information were not necessarily expected to consistently collect it*. (Large teaching hospital)

Eight hospitals had not used these data internally prior to the regulation.

*There was wide variation because it wasn't being reported anywhere and at that point this organization, and many organizations, really weren't using it for purposes to enhance quality and patient experience and the database was not looked at by anyone really*. (Large teaching hospital)

The remaining hospitals had used these data for tailoring interpreter services. Only 11 hospitals had used race data for community outreach, stratifying quality measures, or cultural competency training for staff.

### Implementation of the Regulation

Many hospital executives mentioned concerns from staff and patients during the early phase of implementation. Staff concerns about potentially upsetting patients were cited more frequently than patient concerns. Executives from 17 hospitals mentioned staff concerns that patients might be uncomfortable or reluctant to answer questions about their race and ethnicity.

*They were concerned that patients would be reluctant to answer questions about race, ethnicity, and language... That they would have questions about why, whether it would impact their care, so there was concern about having to ask those types of questions and how patients might respond*. (Large teaching hospital)

*I think people had a discomfort with asking what they perceived as a personal question, and what might be a touchy area for some people*. (Medium teaching hospital)

Although less frequently reported than concern from staff, executives from nine hospitals also reported initial concerns from patients.

*They were a little angry at first because, you know, 'I'm not an American so you don't want to treat me?'... A lot of people were declining at the beginning... Some patients say American no matter what they are. We can tell they are a different ethnic group, but they'll say American. Some people will decline and say, 'we're not telling you, it's none of your business.' Others will give you Heinz 57; they'll give you everything that they have*. (Medium non-teaching hospital)

*Some of the patients perceived that by declaring their ethnicity, they would receive different care, and they didn't mean that in a positive way. Patients don't perceive that to mean it's for us to incorporate their cultural needs into the plan of care, even though that is what we use that information for*. (Medium non-teaching hospital)

In anticipation of these issues, the MHA developed training materials, which were almost uniformly utilized by hospitals.

When asked about feedback from staff and patients after the regulation had been in place for two years, most hospital executives indicated that the process has become routine with fewer issues than anticipated. While 11 hospitals had received some negative feedback from patients, all but one had experienced few complaints or incidents.

*Most provide the information without question; some joke about their ethnicity when asked, and we have had a couple of patients take offense to the question. We had only one patient file a formal complaint about being asked, saying it was none of our business and asked what right we had to ask the question*. (Medium non-teaching hospital)

*We have occasional questions that pop up from patients as to why we're collecting, so on and so forth, but most of the staff are able to address those when they're collecting the information given the training*. (Large teaching hospital)

Only one hospital executive from a non-teaching hospital outside of Boston reported that patients had not yet accepted the change in protocol and obtaining data was still a struggle.

*I don't think they are used to it. They don't like to register to begin with, really. You're pushing a hard button when you ask race, that's for sure*. (Medium non-teaching hospital)

Similarly, executives from only two hospitals reported reservations from staff who sometimes doubted the validity of self-reported data.

*I have actually heard a couple comments of, 'is it truly valid if someone says their ethnicity is white American and clearly they don't appear to be?', but we have to take it at face value*. (Medium non-teaching hospital)

Another hospital representative had not heard of any specific issues, but suspected that staff members were modifying the process to minimize their discomfort about asking potentially intrusive questions. This hospital and several others were monitoring the data collected by individual staff and retraining them when needed.

When asked about problems with the regulation, the most commonly mentioned issue was small numbers in the expanded ethnicity categories. Nearly half of hospitals needed to combine several years of data or collapse many ethnicity categories to perform meaningful analyses. For comparing data across hospitals, one hospital representative noted a lack of standardization when combining ethnic categories.

The removal of a Hispanic category from the race designations was mentioned by executives of six hospitals.

*A very, very big problem... is that somehow, someway Hispanic was eliminated as a race. It is now considered an ethnicity. The Hispanic/Latino population does not know this. So when you ask them what their race is and you give them their choices, they get very upset that they do not have the choice of Hispanic or Latino*. (Medium non-teaching hospital)

*Just not being able to indicate someone's race as Hispanic. They were required to put white or black and some Hispanic people don't consider themselves either*. (Large teaching hospital)

More generally, representatives of seven hospitals indicated that patients remain uncertain how to negotiate the concept of ethnicity.

*The biggest stumbling block we've had, and that we continue to have is, people understanding what the word ethnicity means. That's a big word for a lot of our patients. They don't understand it. They don't understand what it is that we're asking*. (Medium non-teaching hospital)

*When we ask it here at our hospital, if I were to ask you, I would say: 'What do you consider your ethnicity to be? I'm Scottish.' And when I add that 'I'm Scottish' at the end of the sentence, then they know how to answer me back, but when I don't add that they have no idea what to do*. (Medium non-teaching hospital)

### Impact of the Regulation on Hospitals

Prior to the regulation in 2007, eight of the 28 hospitals in our study were not using data on patients' race, ethnicity and language internally, but by 2009 only one hospital was still not using these data. All other hospitals have used these data to describe the racial and ethnic distribution of patients utilizing hospital services. These hospitals have also used these data to refine interpreter services by ensuring the language needs of the hospital population are met through adequate staffing and translation of patient resources. The number of hospitals using the data beyond refining interpreter services increased substantially from 11 to 21 after the regulation, including 13 that were stratifying quality performance measures related to a variety of clinical processes and outcomes. Sixteen hospitals had started using these data for program development and patient outreach.

Hospitals with sufficient minority populations were able to use the granular ethnicity categories to identify disparities within the broad racial classifications. For example, one hospital was stratifying quality measures for Eastern European ethnic groups that had previously been lumped into the 'White' race category. Another hospital was able to separately analyze health outcomes for African Americans, Afro-Caribbeans, and African immigrants, which was not possible when all these groups were simply identified as 'Black'. One large Boston hospital had developed patient outreach programs within specific ethnic groups at several affiliated clinics. Two smaller hospitals outside of Boston were also using data for targeted program development.

*I have a program looking at health care disparities surrounding type II diabetes in Hispanics and we use that data. We're also using it for breast cancer disparities*. (Medium teaching hospital)

*Our maternal-child area has done some significant stuff in terms of the ethnicity and cultural diversity of certain populations that they may be seeing in terms of their beliefs around childbirth, etc. And even post-delivery expectations of their culture, in terms of educating staff*. (Medium non-teaching hospital)

Other uses included stratifying patient satisfaction surveys by race and ethnicity and tailoring staff education and cultural competency training. A full list of examples for how hospitals have used these data is included in Table 2.

**Table 2:** Examples of Data Use By Hospitals

Stratification of Quality Measures (between broad race categories or specific ethnic groups)
• Patient satisfaction surveys
• Readmission rates and length of stay for surgical patients
• Receipt of standard of care for cancer patients
• Access to mental health services
• Diabetes control measures
• Vaccination and cancer screening rates
• Medical errors
• Breast cancer risk and outcomes
Program Development
Identification of prominent ethnic groups to support:
• Targeted cultural competency training for employees
• Focus groups to explore beliefs about childbirth and post-delivery expectations
• Patient outreach to underrepresented populations
Identification of disparities between racial or ethnic groups to support:
• Mobile unit providing free vaccines within neighborhoods with clustered minority populations
• Diabetes care improvement program within community health center
• Colon cancer screening program within community health center

### Barriers to Data Use

Factors mentioned as barriers to data use by all respondent hospitals are included in Table 3. The most commonly cited barrier overall was insufficient resources. This factor was listed as the primary barrier for four of the five DSH hospitals. Another significant theme that emerged was a need for more guidance

**Table 3:** Factors Mentioned as Barriers to Data Use (number of respondents)

• Insufficient resources (11)
• Lack of direction/guidance from the state (10)
• IT data system reformatting and compatibility (7)
• Small numbers within ethnic groups (7)
• Lack of need in context of patient population (4)

from the state about actions hospitals could take with the new data.

I'm not a clinician and I'm not an IT person, so... how does just collecting race and ethnicity help me with the next step? I think there are some steps missing in between that would be helpful ... Some hospitals know what they're doing more on that and others may not, so maybe having some consistent things to tell us to be looking for, I think would be helpful. (Large teaching hospital)

One executive from an academic hospital with a diverse patient population indicated that she wished the regulation had gone further by requiring data collection in all hospital departments, including ambulatory care. Prior to the regulation, this representative was unable to obtain adequate financial support from her institution to track disparities.

*To devote the resources it took to develop that programming and to change things when there are so many other operational issues that had to happen, having a state mandate is what made it happen. It probably never would have happened unless we had a huge grant to get it paid for*. (Medium teaching hospital)

She is now able to address disparities within inpatient, observation unit, and emergency room departments, but is still unable to perform analyses within ambulatory care units, which were not targeted for data collection by the regulation.

Representatives of seven hospitals mentioned continuing issues with information technology systems, such as enabling data systems to "talk with each other" smoothly or technical issues with reporting the data.

*We have the race and ethnicity information on one side, we have the quality information on another. The trouble that we are now having is trying to marry those two pieces of information so that it does become useful*. (Medium non-teaching hospital)

This was an especially common barrier among smaller hospitals, and was frequently described as a burden on hospital resources.

Among the seven hospitals that were using the state-mandated data only for interpreter services or not at all, the most frequently mentioned barrier to use was lack of need within the context of the hospital's patient population, followed by insufficient resources. These seven hospitals were all medium sized, non-teaching hospitals located outside of Boston.

## Discussion

The essential role of accurate data to identify and address racial and ethnic disparities in the quality of health care has become increasingly apparent[4-10]. Efforts to improve the collection of race, ethnicity and language data are being undertaken by the American Hospital Association, America's Health Insurance Plans, and the federal Centers for Medicare and Medicaid Services[5,16,17]. Efforts by health care providers to collect race and ethnicity data are supported by studies that have assessed patients' concerns about these efforts, and proposed solutions to these concerns, including a clear focus on quality improvement and standardized approaches to data collection through state mandates[18-21].

Our interviews with Massachusetts hospital executives provided insights into the role of a new state regulation to establish standardized collection and reporting of race, ethnicity, and language data. These executives reported that prior systems for collecting these data were generally disorganized and ineffective, yielding data of questionable value. Aside from large academic health centers, few hospitals prior to the state regulation were using these data internally except for interpreter services.

Previous research identified barriers encountered by health care organizations implementing more refined methods for collecting race, ethnicity and language data[5,22]. Many of these barriers were mentioned during our interviews, particularly staff concerns about offending patients or causing them psychological discomfort, but these concerns generally subsided as data collection became routine. Only two of 28 hospitals reported frequent complaints from patients. More commonly reported problems with implementing the regulation concerned categorizing Hispanic patients, negotiating the concept of ethnicity, and modifying information systems, all of which were identified as key challenges prior to implementation[13]. Other frequently mentioned problems related to lack of resources for data analysis, and insufficient guidance from the state.

Our study suggests that the regulation had a significant impact on whether and how hospitals were collecting and using race, ethnicity, and language data. Over half of hospitals have begun to use patient race and ethnicity data to identify disparities in quality performance measures for a variety of clinical processes and outcomes and to develop patient services and community outreach programs based on findings from these data.

The hospitals most actively involved in utilizing the data were generally larger teaching hospitals and those with more diverse patient populations. Boston hospitals were also more likely to utilize these data, perhaps because a similar data collection mandate was previously developed and implemented in July 2006 by the Boston Public Health Commission in collaboration with leaders from major Boston hospitals[23]. This regulation included the additional requirement for hospitals to undertake targeted quality improvement efforts when disparities are identified[13]. Hospitals in Boston participated in piloting the earlier city regulation and its associated training materials, which facilitated relatively smooth adoption of the subsequent state mandate. However, several hospital executives mentioned that the state should do more to educate patients about the regulation. A need for guidance to hospitals about using these data was also commonly expressed.

The most significant issue in implementing the collection of race and ethnicity data and subsequently using these data has been information system requirements. A recent IOM report on the collection of race, ethnicity and language data recommends national standards for granular categories of ethnicity that can be rolled-up for reporting and analysis, Hispanic categorization, and multiple response options, which would help to address many of the issues mentioned by participants in our study. Furthermore, the IOM has recommended state-level regulations for data collection, which are easier than federal regulations to enforce, monitor, and tailor for relevance to a state's patient population[4].

This study had some limitations. Our cohort included only 28 of the 78 acute care hospitals in Massachusetts. Respondents were more likely to represent hospitals with residency training programs, those in Boston or the Metrowest area, and those with more than 100 beds. They were also more likely to have been involved in efforts to design and pilot the regulation. In addition, our study was qualitative in nature and based on the perspectives of key hospital executives, but we did not direct observe the process of data collection at participating hospitals or interview staff members who were directly responsible for data collection.

## Conclusions

In response to the Health Information Technology for Economic and Clinical Health (HITECH) Act of 2009, the federal Department of Health and Human Services has issued criteria for the meaningful use of electronic health records,[24] thereby supporting incentives in the Medicare and Medicaid programs for hospitals to adopt standardized procedures for collecting and storing electronic data on patients' race, ethnicity and preferred language[25]. Furthermore, section 4302 of the Patient Protection and Affordable Care Act of 2010 builds upon this foundation of electronic health records, requiring all federally-funded health care programs and surveys to collect self-reported race, ethnicity and language data to enable federal monitoring of health care quality and disparities.

By developing and implementing a statewide regulation for the collection of patients' race, ethnicity and language data by hospitals, the Massachusetts experience provides insight into important policy issues, such as designing tools to support state-mandated data collection, obtaining higher quality data through implementing state regulatory policies, and supporting efforts to reduce disparities through data collection. As other states consider implementing similar regulations to collect race, ethnicity and language data for hospitalized patients, they can learn from the experience of Massachusetts hospitals that have been required to collect these data since 2007. The response of Massachusetts hospitals to the mandate demonstrates that regulating the collection of these data can be an effective approach to promote performance monitoring and quality improvement, thereby setting the stage for federal standards and incentive programs to eliminate racial and ethnic disparities in the quality of health care.

## Competing interests

The authors declare that they have no competing interests.

## Authors' contributions

SJ was involved in obtaining funding, study design, data collection and analysis, and writing of the manuscript. RT was involved in study design, data analysis, and editing of the manuscript. RMW was involved in the study design and editing of the manuscript. JZA was involved in obtaining funding, study design, data analysis, writing and editing of the manuscript, and is the corresponding author and will act as guarantor for the work. All authors have read and approved the final manuscript.

## Pre-publication history

The pre-publication history for this paper can be accessed here:

http://www.biomedcentral.com/1472-6963/10/352/prepub

## Supplementary Material

Additional file 1 **Massachusetts Department of Public Health Race-Ethnicity and Language Preference Instrument**. The data collection tool provided to hospitals by the Massachusetts Division of Health Care Finance and Policy to enable standardized collection of race, ethnicity and language preference from patients. Click here for file

Additional file 2 **Semi-structured Interview Guide**. The outline used to direct the discussion between the interviewer and hospital representatives. Click here for file
