# Supplementary material for: Responses of Massachusetts hospitals to a state mandate to collect race, ethnicity and language data from patients: a qualitative study
Source: BMC Health Serv Res. 2010 Dec 31;10:352. doi: 10.1186/1472-6963-10-352 (PMC3022878; doi:10.1186/1472-6963-10-352)
Supplement: Additional file 1 — Massachusetts Department of Public Health Race-Ethnicity and Language Preference Instrument. The data collection tool provided to hospitals by the Massachusetts Division of Health Care Finance and Policy to enable standardized collection of race, ethnicity and language preference from patients. [file 1472-6963-10-352-S1.DOC]

| MITT ROMNEY  Governor  KERRY HEALEY  Lieutenant Governor  TIMOTHY R. MURPHY  Secretary  PAUL J. COTE, JR.  Commissioner | The Commonwealth of MassachusettsExecutive Office of Health and Human ServicesDepartment of Public Health250 Washington Street Boston, Massachusetts 02108-4619 |
| --- | --- |

## **MDPH Race-Ethnicity and Language Preference Instrument,**

## **November 28, 2006 - REVISED**

## ***Introduction: In order to guarantee that all patients receive the highest quality of care and to ensure the best services possible, we are asking all patients about their race, ethnicity, and language.***

1. **Are you Hispanic/Latino/Spanish?**

- Yes ❑ No

**2. What is your ethnicity? (You can specify one or more)**

| - African - African American - American - Asian - Asian Indian - Brazilian - Cambodian - Cape Verdean - Caribbean Island - Central American (not otherwise specified) - Chinese - Columbian - Cuban - Dominican - Eastern European - European | - Filipino - Guatemalan - Haitian - Honduran - Japanese - Korean - Laotian - Mexican, Mexican American, Chicano - Middle Eastern - Portuguese - Puerto Rican - Russian - Salvadoran - South American (not otherwise specified) - Vietnamese - Other Ethnicity - Unknown/not specified |
| --- | --- |

**3. What is your race? (You can specify one or more)**

- American Indian/Alaska Native
- Asian
- Black/African American
- Native Hawaiian or other Pacific Islander
- White
- Other Race
- Unknown/not specified

**4. In what language do you prefer to discuss health-related concerns?**

| - English - Spanish - Portuguese - Cape Verdean Creole - Haitian Creole - Khmer - Vietnamese | - Somali - Arabic - Albanian - Chinese (specify dialect___________) - Russian - Other (specify____________) |
| --- | --- |

**5. In what language do you prefer to read health-related materials? _______________**
