# Supplementary material for: Responses of Massachusetts hospitals to a state mandate to collect race, ethnicity and language data from patients: a qualitative study
Source: BMC Health Serv Res. 2010 Dec 31;10:352. doi: 10.1186/1472-6963-10-352 (PMC3022878; doi:10.1186/1472-6963-10-352)
Supplement: Additional file 2 — Semi-structured Interview Guide. The outline used to direct the discussion between the interviewer and hospital representatives. [file 1472-6963-10-352-S2.DOC]

**Semi-structured Interview Guide**

Part One: Knowledge of the regulation(s)

I would like to begin by asking about your familiarity with the regulation.

1. When did you first become aware of the Massachusetts (and Boston) regulation(s) for the collection of race and ethnicity (and language) data?
2. What was your understanding of the purpose behind the regulation(s)?
3. How was/were the regulation(s) perceived within your hospital?

Part Two: Prior to the regulation(s)

I will now ask questions regarding data collection at your hospital prior to the regulation(s).

1. Before the implementation of the state (and city) regulation(s), did your hospital collect any race, ethnicity, or language data about your patients? If yes:
   1. What type of data was collected?
      1. Race
      2. Ethnicity
      3. Language
      4. A combination
      5. Other (please specify)
   2. Are you familiar with the types of data categories that were used?
   3. Are you familiar with how the information was collected?
   4. Was the information you collected used in any way? If yes, please elaborate.

Part Three: Implementing the regulation(s)

This next set of questions addresses the implementation of the regulation(s).

1. Did compliance with the regulation(s) require the hospital to train staff in collecting patient race, ethnicity and language data? If yes:
   1. Approximately how many staff had to be trained?
2. Prior to implementing data collection, were you aware of any concerns from either staff or patients about collecting the data? If yes, please elaborate.
3. Now that the regulations have been in force since mid-2007,
   1. What feedback has your hospital received from staff about collecting the data, if any?
   2. What feedback has your hospital received from patients about collecting the data, if any?
4. Have any aspects of the regulation(s) been particularly problematic? If yes, please elaborate.

Part Four: Impact of the regulation(s) within the hospital

The final set of questions address the impact of the regulation(s) on your institution.

1. Are you currently using, reporting or analyzing the race, ethnicity or language data in any way within your hospital in addition to reporting it to the state or city?

If yes, I’d like to ask your some more detail about how you have used this data:

1. Have the data been used to describe the race/ethnicity of those utilizing hospital services?
2. Have these data been used to describe the primary language of those using hospital services?
3. Have the data been used to stratify performance or quality measures? If yes, which ones?
   1. Were disparities found in any of these measures? If yes, please elaborate.
4. Who is the main audience to whom data analyses are presented?
5. How frequently are these data presented to the relevant audience?
6. Does your hospital have a disparities committee?
7. Does your hospital’s mission statement include a commitment to addressing disparities?
8. Are the data publicly available through the hospital’s website?
9. Have the data been used to add or plan new services for patients? If yes, please elaborate.
10. Have the data been used to develop programs or projects intended to address disparities?
11. Are these data now included in routine quality monitoring?
12. Have any of the following presented challenges to your use of the data?
    1. Small numbers within race/ethnicity/language categories
    2. Lack of resources/analysts
    3. Doubts about quality of data
    4. Lack of perceived benefit/necessity
    5. Other (please elaborate)
13. Do you think your hospital would have collected these data in the absence of the city and state mandates?
14. Do you think the mandates are an effective way to address health disparities?

If no:

1. Do you have any plans to use, analyze or report the race, ethnicity or language data internally in the future?
2. Do any of the following present challenges to your use of the data?
   1. Small numbers within race/ethnicity/language categories
   2. Lack of resources/analysts
   3. Doubts about quality of data
   4. Lack of perceived benefit/necessity
   5. Other (please elaborate)
3. Does your hospital’s mission statement include a commitment to addressing disparities?
4. Does your hospital have a disparities committee?
5. Do you think your hospital would have collected these data in the absence of the city and state mandates?
6. Do you think the mandates are an effective way to address health disparities?

Is there anything else you would like to add?
